# Embryonic manipulations shape life-long, heritable stress responses through complex epigenetic mechanisms: a review

**DOI:** 10.3389/fnins.2024.1435065

**Published:** 2024-07-19

**Authors:** Tatiana Kisliouk, Padma Malini Ravi, Tali Rosenberg, Noam Meiri

**Affiliations:** ^1^Institute of Animal Science, Agricultural Research Organization, Volcani Center, Rishon Leziyyon, Israel; ^2^Department of Animal Science, The Robert H. Smith Faculty of Agriculture, Food and Environment, The Hebrew University of Jerusalem, Rehovot, Israel

**Keywords:** chick, epigenetics, hypothalamus, stress, embryonic

## Abstract

Enhancing an organism’s likelihood of survival hinges on fostering a balanced and adaptable development of robust stress response systems. This critical process is significantly influenced by the embryonic environment, which plays a pivotal role in shaping neural circuits that define the stress response set-point. While certain embryonic conditions offer advantageous outcomes, others can lead to maladaptive responses. The establishment of this response set-point during embryonic development can exert life-long and inheritable effects on an organism’s physiology and behavior. This review highlights the significance of multilevel epigenetic regulation and the intricate cross-talk among these layers in response to heat stress during the embryonic period, with a particular focus on insights gained from the avian model.

## Introduction

In an individual, there are probably initial stress-response set points that are determined genetically (Daskalakis et al., 2013). Nevertheless, the intricate interplay of genetic and epigenetic factors during embryonic development further shapes the trajectory of stress response systems. This interweaving of genetic predispositions and environmental cues establishes a foundation for an organism’s lifelong stress coping mechanisms. On one hand, poor embryonic development may lead to adult adversities (Galler and Rabinowitz, 2014; O’Donnell and Meaney, 2017). On the other hand, the embryonic environment can have a beneficial effect on improving stress tolerance (Pribenszky et al., 2010; Rosenberg et al., 2022). Hence, understanding and optimizing embryonic conditions hold promise for not only individual survival but also for the transmission of adaptive traits across generations. While the life-long effects of the embryonic environment are clear and apparent, the mechanisms underlying the adjustment of the set-point and its epigenetic inheritance are only starting to be revealed.

### The chick model serves as an invaluable tool for investigating the life-long and heritable effects of embryonic heat stress

To investigate the intricate mechanisms underlying the long-lasting impact of stress during embryonic development, a robust research model becomes imperative. While manipulating embryos in mammals poses challenges, often reliant on maternal factors influenced by abnormal pregnancies like maternal addiction (Bara et al., 2021), or over nutrition (Sarker et al., 2019) the avian chick emerges as an ideal candidate. Its ease of manipulation and pharmacological intervention, along with the lack of maternal dependence in these precocial birds, and immediate post-hatch learning abilities, render chicks an optimal system for studying developmental responses to environmental stressors (Frésard et al., 2013; Yahav and Brake, 2014; Bednarczyk et al., 2021). Moreover, their readily measurable post-hatch behavioral repertoire, alongside a hypothalamic thermal control system very similar to that of mammals, which resides in the preoptic area/hypothalamus (PO/AH), enhances their suitability for comprehensive research (Tan and Knight, 2018; Bohler et al., 2021).

In recent years, both our team and others have pioneered the use of a chick model to investigate responses to embryonic and early-life thermal stress (Loyau et al., 2016; Cramer et al., 2019; Rosenberg et al., 2020, 2021, 2022; Iraqi et al., 2024). These studies have indicated that enhancing thermotolerance in poultry is achievable through exposure to elevated ambient temperatures during embryogenesis. This embryonic heat conditioning (EHC) involves subjecting fertilized eggs to cyclic fluctuations in incubation temperature during days 7–16 of the incubation, resulting in increased thermal resilience (Piestun et al., 2008, 2009; Loyau et al., 2015). Moreover, our research has elucidated that the ‘memory’ of thermal stress response is governed by the PO/AH and is intricately regulated by multilevel epigenetic mechanisms (Rosenberg et al., 2020, 2021, 2022). Furthermore, EHC beneficial effect on heat resilience has broader consequences, since in recent years, it was shown that conditioning to one stressor can induce resilience to another, different one. For example, thermal conditioning induces neuroprotection against traumatic brain injury (Umschweif et al., 2014). This phenomenon, known as cross-tolerance, is attributed to tissue-specific activation of generic stress signaling (Horowitz, 2017). Indeed, we showed in addition to thermotolerance, EHC induces cross-tolerance with the immune system, attenuating hypothalamic inflammation, following lipopolysaccharide (LPS) challenge, manifested by reduced febrile response, reduced expression of LPS induced immune related genes such as TNF factor (LITAF), NFκB and IL6, and less nuclear localization and activation of NFκB in the hypothalamus (Rosenberg et al., 2021).

### Multilevel epigenetic regulation of embryonic heat stress effects

The different layers of epigenetic markers implicated in regulating embryonic gene expression for attaining thermal resilience and the broader effect of stress cross tolerance encompasses DNA CpG methylation, histone post-translational modifications, microRNA (miRNA), and chromatin topologically associating domains (TADs) organization. The first layer which shapes the neural network during embryonic development involves histone post translational modification which is thought to affect the tightness of the chromatin folding (Armeev et al., 2021). During regular development, histone post-translational modifications play pivotal roles in both peripheral development and neurogenesis. In the context of embryonic heat stress in chicks, maternal heat stress profoundly affects the embryo peripherally and in the central nervous system. In the periphery, embryonic heat stress induces hyperacetylation of histone H3 at lysine 9 (H3K9) in the liver, leading to increased embryonic mortality. These adverse outcomes can be alleviated through maternal zinc supplementation (Zhu et al., 2017). During neurogenesis, studies have revealed that histone post-translational modifications, specifically methylation and acetylation of histone H3 at lysine 27 (H3K27), play a role in the embryonic reprogramming of cells in the retinal pigment epithelium (RPE) into retina progenitor cells, which subsequently differentiate into major retinal cell types (Luz-Madrigal et al., 2020). Furthermore, Jiao et al. (2013) demonstrated the involvement of H3 acetylation and methylation on the avian promoter of cPOUV, a crucial pluripotency-related factor, during the initial phases of chick embryonic development. In the context of heat stress, in the hypothalamus, chromatin immunoprecipitation sequencing (ChIP-seq) studies of histone H3 at lysine 4 (H3K4) and H3K27 methylation of thermally conditioned embryonic chicks demonstrated peaks around genes associated with metabolic processes, gene expression regulation, neuronal development, and immunity-related functions (David et al., 2019). Studying the multilevel epigenetic regulation of the cross-tolerance effect of EHC on inflammation resilience in the hypothalamus. As a test case, we focused on the epigenetic regulation of IL6 cytokine gene, particularly the first intron, identified as an enhancer region. Changes in this intron due to EHC showed increased H3K27me3, a repressive translational modification. This histone modification occurred during embryonic conditioning and persisted into later life, correlating with a reduced inflammatory and febrile response following lipopolysaccharide (LPS) induction (Rosenberg et al., 2021). Furthermore, correlating with the increase in H3K27me3, we observed a 50% increase in the mRNA expression of enhancer of zeste 2 polycomb repressive complex 2 subunit (EZH2), a known catalyzer of methyl group addition to H3K27, during EHC compared to chicks incubated under regular conditions (Kuzmichev et al., 2002; Rosenberg et al., 2021).

The second layer involves alteration in DNA CpG methylation. DNA methylation is described in correlation with heat stress in animals ranging from *C. elegans* (Xu et al., 2023), to humans (Murray et al., 2022). DNA CpG methylation stands as one of the most extensively studied epigenetic modifications. The embryonic development of chicks offers compelling evidence of an increasing genomic methylation pattern, pivotal for orchestrating transitions across various embryonic stages (Liu et al., 2019; Ju et al., 2023). Notably, during the differentiation of the chick embryonic retina from quiescent cells, a significant increase in promoter methylation is observed (Luz-Madrigal et al., 2020). This methylation pattern, indispensable for normal developmental processes, undergoes modification under EHC. Maternal heat stress has been shown to reduce chick hatchability, coinciding with heightened methylation on the manganese superoxide dismutase (MnSOD) promoter—a phenomenon mitigated by thermal conditioning or maternal manganese supplementation (Zhu et al., 2017). When the embryo in the egg is exposed to high ambient temperature, embryonic development influences DNA methylation of heat shock proteins in brain tissue (Vinoth et al., 2018). In accordance with the phenotypic impact of EHC, which confers lifelong and potentially heritable thermal resilience, we have identified transcriptional changes in EHC-related gene expression within the PO/AH (Rosenberg et al., 2021).

Delving deeper into DNA methylation dynamics unveils the crucial roles played by the writers and erasers of this modification. DNA methylation is orchestrated by a distinct family of enzymes known as DNA methyltransferases (DNMTs). While DNMT3a and DNMT3b are primarily responsible for *de novo* methylation, DNMT1 safeguards the methylation pattern during DNA replication. Throughout embryonic development, all three DNMTs exhibit heightened expression levels, which taper off upon terminal differentiation, except in post-mitotic neurons where DNMT expression remains notable and thought to play a pivotal role in neuronal network organization (Moore et al., 2013; Lyko, 2018; Antunes et al., 2019).

DNA demethylation mechanisms encompass both passive processes occurring during DNA replication and active demethylation, and are facilitated by the ten-eleven translocase (TET) enzyme family comprising TET1-3 members and thymine DNA glycosylase (TDG). TET enzymes catalyze the oxidation of 5-methylcytosine (5mC) to 5-hydroxymethylcytosine (5hmC), now recognized as the sixth DNA base (Kohli and Zhang, 2013; Dean, 2014). Subsequently, 5hmC can undergo further conversion into 5-formylcytosine (5fC) and 5-carboxylcytosine (5caC), serving as intermediates in an active demethylation pathway that ultimately replaces 5mC with cytosine (Wu and Zhang, 2017). It has been shown that TET enzymes mediated response in environmental stress and stress-related psychiatric diseases (Xia et al., 2023).

Notably, EHC of chicks coincides with an upregulation of TET2 mRNA expression and a decrease in DNMT3B expression. The Enhanced TET activity leads to enhanced mRNA expression (Sabino et al., 2022). In ovo inhibition of TET activity results in both general and specific downregulation of 5hmC, impairing the EHC driven heat resilient phenotype later in life (Rosenberg et al., 2020). Additionally, differential expression of DNMTs is evident in liver and muscle tissues of conditioned chick embryos (Li et al., 2016).

Analysis of the CpG pattern of IL6 intron 1 in cross tolerance, revealed a reduction in hydroxymethylation together with increased binding of NFκB (Rosenberg et al., 2021). Hydroxymethylation that occurred during EHC, was driven by induced expression of TET family enzymes. Reversing this effect, in ovo during conditioning, with bis-2-(5-phenylacetamido-1,3,4-thiadiazol-2-yl) ethyl sulfide, completely blocked both TET activity and heat resilience (Rosenberg et al., 2020).

Another layer of epigenetic regulation of heat stress responses is provided by miRNAs. miRNAs are small, highly conserved non-coding RNA molecules that regulate gene expression post-transcriptionally by binding to the 3′ untranslated region of mRNA, thereby influencing their stability and expression levels (Pritchard et al., 2012). Numerous studies have identified several miRNAs that exhibit differential expression in response to heat stress across various tissues of livestock, including the liver, muscle, and blood (Raza et al., 2021; Jaglan et al., 2024). These miRNAs play pivotal roles in regulating heat shock protein synthesis, antioxidant defense mechanisms, and other stress response pathways.

For instance, in a model of early life heat stress in chicks, miR-138 was demonstrated to be involved in enhancing heat resilience (Kisliouk et al., 2014). Moreover, to illustrate the multi-layered epigenetic regulation and the cross-talk between the different layers, underlying the establishment of thermal tolerance, it was revealed that miR-138 impacts the expression of EZH2, which in turn affects histone methylation, thereby influencing thermotolerance acquisition (Kisliouk et al., 2011).

Applying a similar approach to elucidate the epigenetic regulation of gene expression at multiple levels, our research unveiled that during the embryonic period in eggs under heat conditioning, miR-26a can modulate the expression pattern of EZH2, as previously discussed, and serves as a mediator of H3K27me2 methylation in the long-term effects of environmentally induced heat stress responses, as highlighted in a study by Rosenberg et al. (2021).

### Heredity of heat stress response pattern

The debate surrounding the heritability of behavioral traits persists, heightened by reports suggesting that the methylome is erased in sperm, thus making inheritance technically challenging to explain. However, recent research has shed light on the fact that specific loci in sperm DNA escape the erasure process and transmit developmental information to the offspring. *In ovo* heat conditioning offers a unique perspective into the fundamental question of trait heredity (Lismer and Kimmins, 2023). While transgenerational epigenetic effects induced by heat stress are well-documented in plants (Perrella et al., 2022), research on animals remains limited, primarily focusing on species such as Drosophila, *Caenorhabditis elegans*, and Artemia (Houri-Zeevi et al., 2020). Furthermore, studies on transgenerational responses to heat stress in vertebrates are scarce, with some investigations focusing on factors like cattle progeny fertility (Kasimanickam and Kasimanickam, 2021).

In a study on Japanese quails, thermal manipulation across four generations demonstrated significant effects on hatchability, body weight, and the weight of laid eggs. Importantly, some of these effects intensified across generations, with both body weight and egg weight showing transgenerational transmission (Vitorino Carvalho et al., 2023).

In our exploration of the heritable effects of EHC, we have successfully demonstrated the enduring nature of both thermal and immune resilience acquired through such conditioning, as evidenced in the subsequent generation (Rosenberg et al., 2022). Notably, we observed differential DNA methylation patterns in the PO/AH between offspring from fathers subjected to either control conditions or EHC (Rosenberg et al., 2022).

Moreover, we postulate that the repertoire of genes expressed in the offspring of EHC chicks may be influenced by an additional layer of epigenetic organization, contributing to the heritability of heat stress-related traits. This epigenetic layer involves the three-dimensional organization of chromatin (Rosenberg et al., 2022). Such topological organization encompasses the megabase-scale folding of chromatin into nuclear compartments and self-interacting units known as topologically associated domains (TADs; Rowley and Corces, 2018). The orchestration of large-scale 3D chromatin organization is facilitated by key architectural proteins, including the CCCTC-binding factor (CTCF), which delineates chromatin loops and establishes topological boundaries (Xiang and Corces, 2021).

In offspring of EHC treated fathers, we identified 110 differentially expressed genes that were associated with altered methylation, predominantly on enhancers. Gene-ontology analysis shows pathways associated with immune response, chaperone mediated protein folding and stress response. For the proof of concept, we focused on HSP25 and SOCS3, modulators of heat and immune responses, respectively. The chromosome conformational capture (3C) assay showed that the promoters of certain genes interacted with methylated enhancer regions, especially at CTCF binding sites. This means that gene expression was linked to these methylation patterns. Increased binding of CTCF was seen in both highly and lowly methylated CpG sites (DMSs), indicating that both activators and repressors are involved (Rosenberg et al., 2022).

## Conclusion and future perspectives

The questions related to the heredity of heat stress-related traits remain unresolved at both behavioral and biochemical levels. On the phenotypic level, several questions persist: How many generations are affected by EHC? What are the levels of decay in the stress response over time? What is the potential impact of reinforcement in subsequent generations on the intensity and duration of stress responses?

On the epigenetic level, it is evident that all components mentioned in this review interact and influence each other, ultimately determining the precise level of gene expression responsible for the stress response. However, the specific order of these interactions and the necessity of each component in the heredity process remain unclear. Additionally, emerging epigenetic regulatory elements, such as long non-coding RNAs, require further exploration to fully understand their roles.

**Figure 1:**
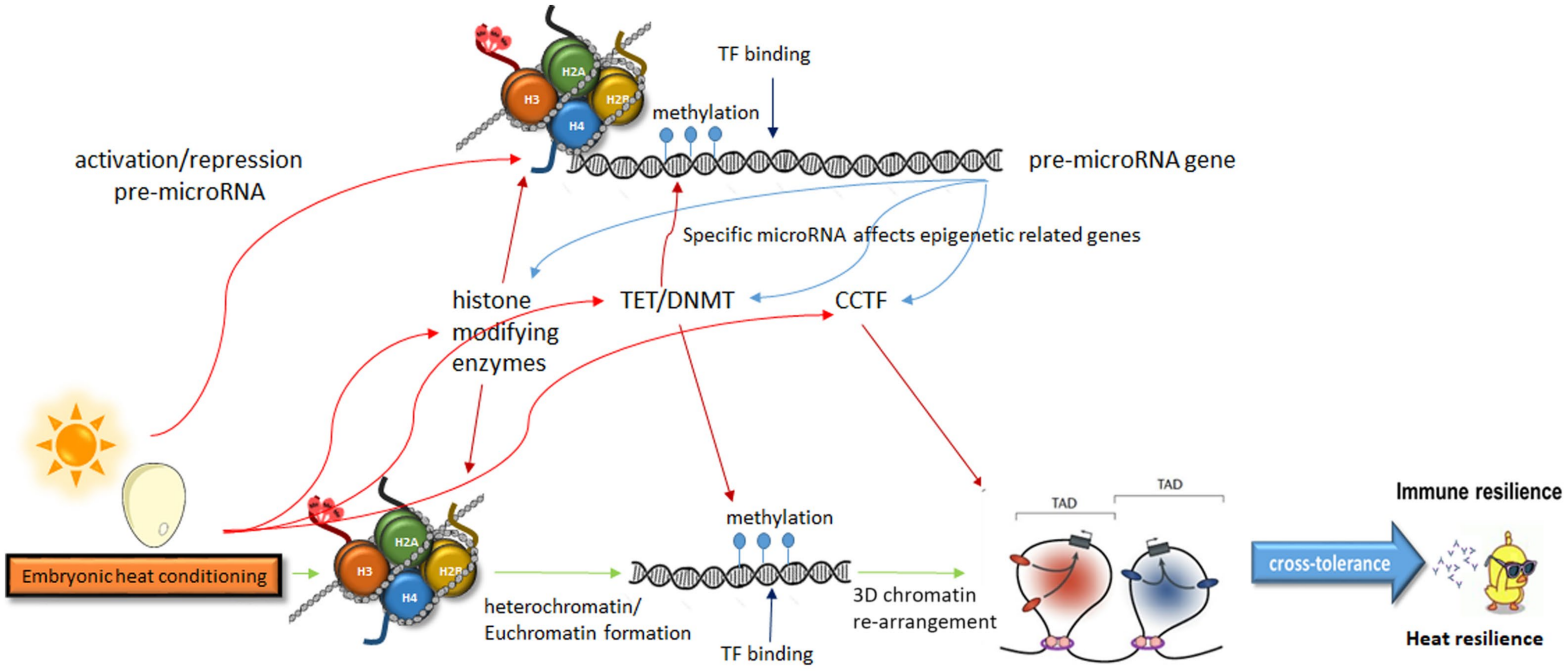
A model describing multiple levels of crosstalk in epigenetic modulation leading from embryonic heat conditioning to heat and immune resilience. Embryonic heat conditioning affects the expression of genes related to epigenetic regulation, influencing neuronal networks that govern stress and immune responses. These include histone modifiers, DNA methylation modulators, and enzymes regulating chromatin organization. Specific pre-miRNAs, also modulated by these enzymes, impact the expression of epigenetic-related genes. This intricate interplay of epigenetic regulation fine-tunes stress responses, leading to immune and heat resilience. TAD, topologically associating domain; TF, transcription factor; CCTF, CCCTC-Binding factor; TET, Ten-eleven translocation; DNMT, DNA-methyltransferase.

The acknowledgment of the heritability of behavioral traits is gaining traction within the scientific community. Emerging evidence increasingly points toward the involvement of epigenetic regulation in such heritability. Given the rapid influence of physiological responses on behavior, which can have significant evolutionary implications, the regulation of this heredity must be exceptionally precise. Consequently, the epigenetic regulation appears to be intricately orchestrated, likely encompassing multiple levels of crosstalk between various epigenetic modulators. The order of epigenetic events is unclear, and the interactions between them affect each other. Therefore, here we did not commit to a specific sequence as it might change under different circumstances. Consequently, conditioning likely affects all the epigenetic steps simultaneously (Figure 1).

The intricate network of epigenetic mechanisms implicated in the adaptive response to heat stress offers invaluable insights into potential intervention targets aimed at bolstering thermal tolerance in diverse organisms, including livestock. Understanding and manipulating these mechanisms hold promise for improving resilience to environmental challenges and enhancing overall adaptability.

These perspectives highlight the need for continued research to unravel the complex interplay of epigenetic factors governing the inheritance of heat stress responses. Such insights will not only deepen our understanding of stress heredity but also pave the way for innovative strategies to enhance the resilience and adaptability of organisms in the face of environmental challenges.

## Author contributions

TK: Writing – review & editing. PR: Writing – review & editing. TR: Writing – review & editing. NM: Writing – original draft, Writing – review & editing.
